# Supplementary material for: Pheromone representation in the ant antennal lobe changes with age
Source: Curr Biol. Author manuscript; Available in PMC 2024 Jul 24. (PMC11265976; doi:10.1016/j.cub.2024.05.031)
Supplement: MMC1 [file NIHMS1997610-supplement-MMC1.pdf]

Current Biology, Volume *34*

Supplemental Information

Pheromone representation in the ant antennal lobe changes with age

Taylor Hart, Lindsey E. Lopes, Dominic D. Frank, and Daniel J.C. Kronauer

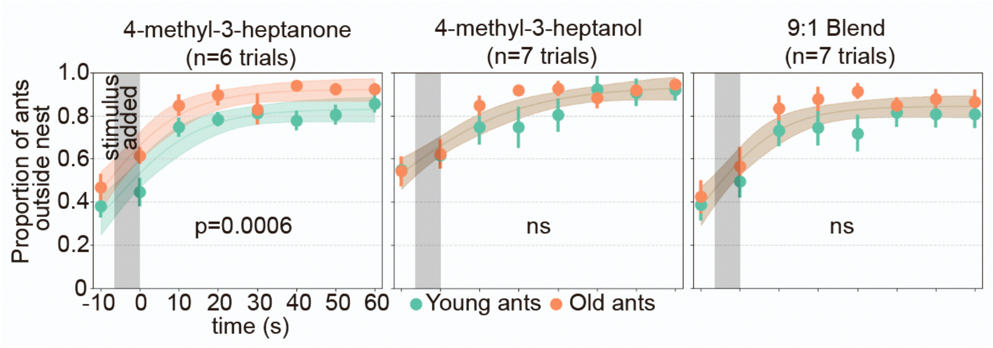

Figure S1. Colony alarm bioassay data for individual alarm pheromone stimuli. Related to Figure 1. Data points show mean±SEM. Raw data were fitted with logistic regressions, with ribbons showing 95% CI. P values relate to whether the single curve model was rejected in favor of separate curves for young and old ants (extra sum-of-squares F test). For 4-methyl-3-heptanone, a model with separate curves was preferred. For 4-methyl-3-heptanol and the blend, similar trends were observed, but single curve models were preferred. See Data S1 for behavioral data. See Table S1 for details on the statistical analyses. Grey bars show the time window when the stimulus was added to the arena. ns: $p>0.05$.

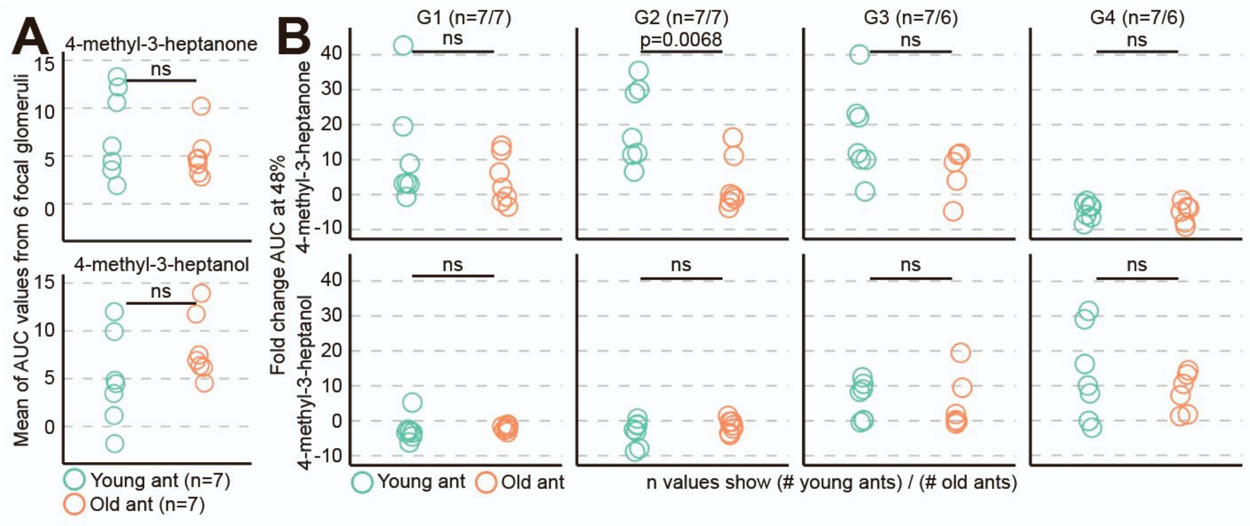

Figure S2. Additional quantification of alarm pheromone responses in the focal glomeruli. Related to Figure 3. A) AUC values from alarm pheromone responses in all six focal glomeruli (shown individually in (B) and Figure 2D) were averaged for each individual and then compared between age cohorts (Welch's T-tests). **B)** Area under the curve values calculated from the calcium response time series data shown in Figure 3B. Statistical test results are shown here again (Welch's T-tests). Odorants were presented at 48% concentration v/v. See Data S2 for calcium imaging data. ns: $p>0.05$.

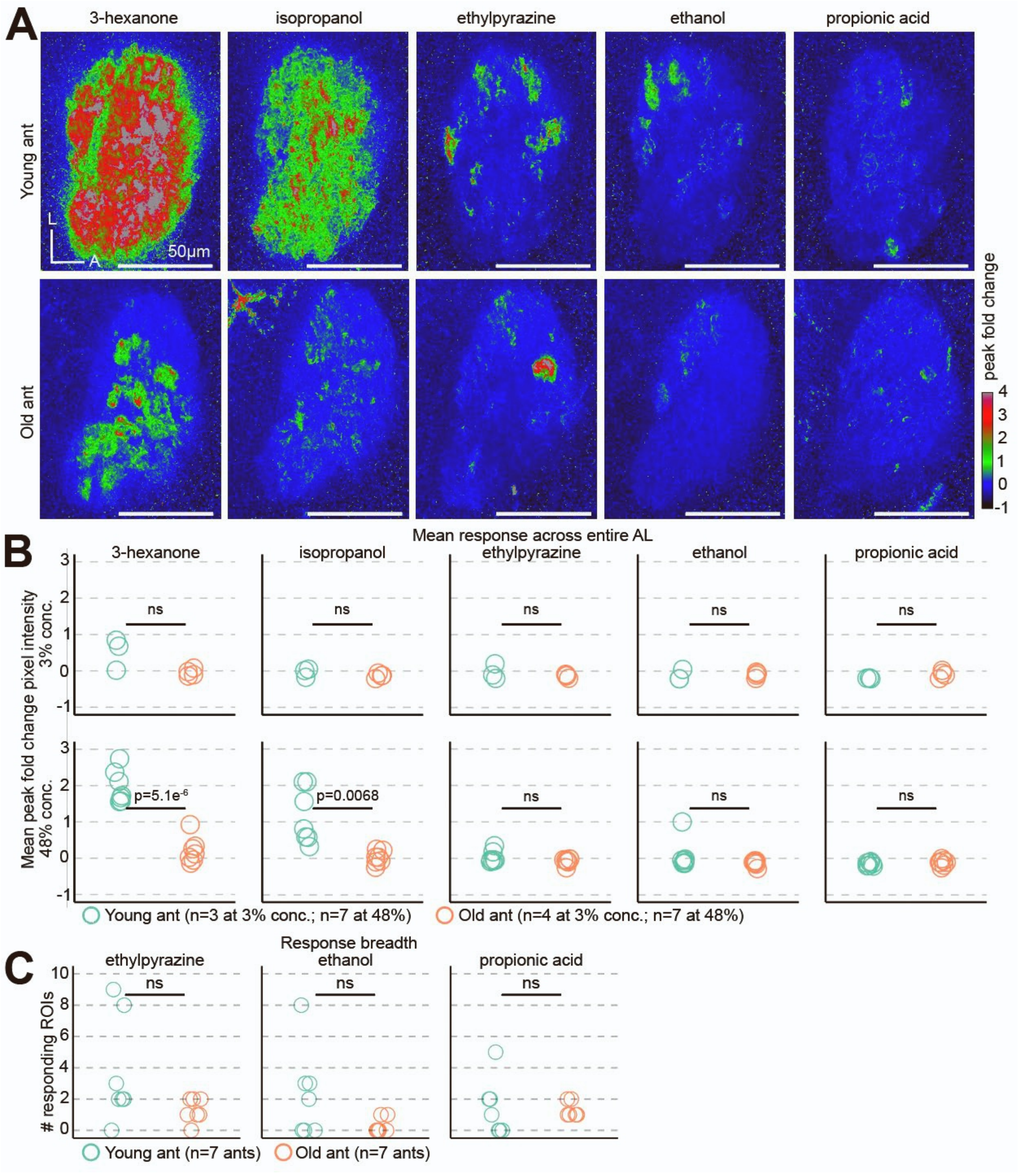

Figure S3. General odorant responses are modulated with age. Related to Figure 4. A) Exemplar response maps for the five general odorants, showing max Z-projections of the peak fold change across the entire right AL, from a young ant (top) and an old ant (bottom), both at

48% odor concentration v/v. **B)** Mean pixel intensities across ROIs of the entire AL response maps, at 3% (top) and 48% (bottom) odor concentration v/v. Responses were compared across age cohorts (Welch's T-tests). **C)** Comparison of the number of responding ROIs in young ants vs. old ants at 48% odor concentration v/v, excluding broadly represented odorants (Wilcoxon tests). See Data S2 for calcium imaging data. L: lateral; A: anterior. ns: $p>0.05$.

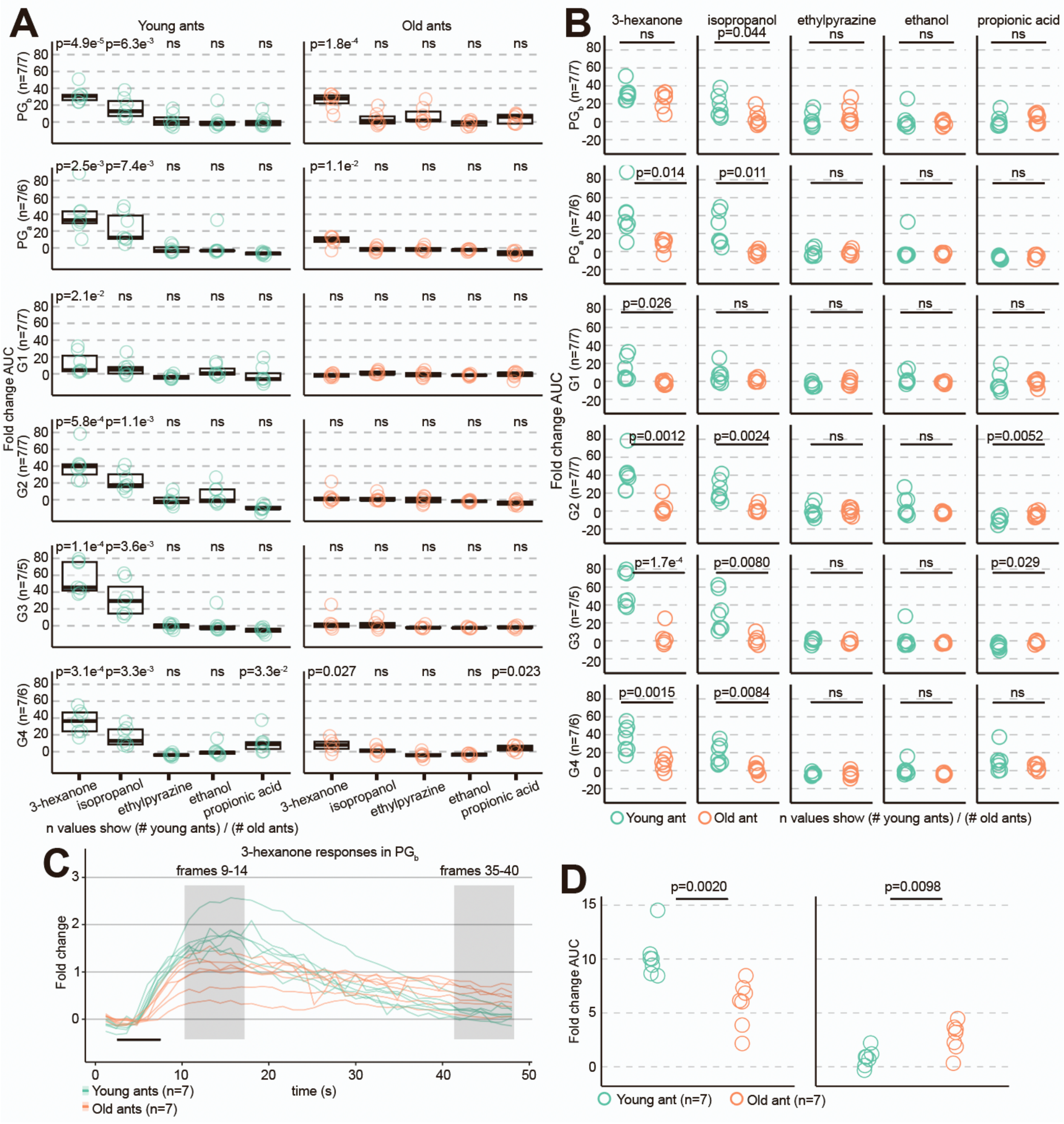

Figure S4. Additional quantification of general odorant responses in PG_b and PG_a. Related to Figure 4. A) Quantification of odor-evoked responses in the six focal glomeruli in young (left) and old (right) ants. Data show the area under the curve (AUC) of the time series data shown in Figure 4. Boxes contain the first to third quartiles and thick lines show the median. Values were compared against the null expectation of zero excitation (one-sided, one-sample

Welch’s T-tests). **B)** Comparison of AUC values between age cohorts. The same statistical tests shown in Figure 4 are shown here again (Welch's T-tests). **C)** Calcium response time series in PG_b from individual ants stimulated with 3-hexanone, to show the effect of age on temporal dynamics of the response. Each line shows the mean of three trials from the same ant. Two time windows of interest are highlighted with grey rectangles. **D)** Mean AUC values were calculated from the two time windows highlighted in (C). For frames 9-14, containing the peak of the response, AUC values were significantly greater in 14 day old (“young”) ants compared to 60 day old (“old”) ants (left, Welch’s T-test). For the final frames of the recordings (frames 35-40), the opposite relationship was found (right, Welch’s T-test). Odorants were presented at 48% concentration v/v. See Data S2 for calcium imaging data. ns: $p>0.05$.

Data	Preferred model (p-value)	F (DFn, DFd)	Different curves (best fit; 95% CI)			One curve (best fit; 95% CI)		
			YM (log) / B0 (quad)	Y0 (log) / B1 (quad)	K (log) / B2 (quad)	YM (log) / B0 (quad)	Y0 (log) / B1 (quad)	K (log) / B2 (quad)
Alarm pheromone stimuli, comparing stimuli (Figure 1B)	One curve for all datasets (0.052)	2.144 (6, 151)	0.876 (0.832 to 0.927) 4-methyl-3-heptanone; 0.936 (0.871 to 1.101) 4-methyl-3-heptanol; 0.845 (0.789 to 0.914) blend	0.593 (0.541 to 0.646) 4-methyl-3-heptanone; 0.660 (0.611 to 0.711) 4-methyl-3-heptanol; 0.587 (0.515 to 0.665) blend	0.0948 (0.063 to 0.135) 4-methyl-3-heptanone; 0.057 (0.038 to 0.103) 4-methyl-3-heptanol; 0.103 (0.026 to 0.098) blend	0.881 (0.849 to 0.919)	0.614 (0.580 to 0.650)	0.084 (0.062 to 0.111)
Vehicle control, comparing age (Figure 1C)	One curve for all datasets (0.056)	2.617 (3, 90)	0.352 (0.252 to 0.453) young; 0.465 (0.403 to 0.527) old	0.001 (-0.002 to 0.004) young; -0.001 (-0.003 to 0.0004) old	3.436e-5 (-1.099e-4 to 1.786e-4) young; 4.284e-6 (-8.500e-5 to 9.357e-5) old	0.408 (0.349 to 0.468)	-3.618e-4 (-0.002 to 0.0014)	1.932e-5 (-6.646e-5 to 1.051e-4)
Pooled alarm pheromone stimuli, comparing age (Figure 1C)	Different curve for at least one dataset (<1.0e-4)	8.006 (3, 314)	0.858 (0.808 to 0.939) young; 0.914 (0.883 to 0.947) old	0.571 (0.527 to 0.617) young; 0.663 (0.626 to 0.703) old	0.070 (0.042 to 0.106) young; 0.099 (0.074 to 0.128) old	0.882 (0.853 to 0.914)	0.617 (0.587 to 0.649)	0.085 (0.065 to 0.109)
4-methyl-3-heptanol, comparing age (Figure S1)	One curve for all datasets (0.122)	1.979 (3, 106)	0.995 (lower bound: 0.847; upper bound could not be determined) young; 0.936 (0.887 to 1.002)	0.632 (0.561 to 0.702) young; 0.692 (0.637 to 0.752) old	0.036 (0.005 to 0.090) young, 0.084 (0.049 to 0.131) old	0.937 (0.879 to 1.056)	0.660 (0.615 to 0.707)	0.060 (0.031 to 0.097)
4-methyl-3-heptanone,	Different curve for at	6.377 (3, 90)	0.830 (0.777 to 0.898) young;	0.534	0.095	0.876	0.598	0.095

comparing age (Figure S1)	least one dataset (6.0e-4)		0.921 (0.872 to 0.983) old	(0.470 to 0.600) young; 0.662 (0.602 to 0.730) old	(0.057 to 0.145) young; 0.097 (0.058 to 0.151) old	(0.836 to 0.922)	(0.551 to 0.647)	(0.065 to 0.132)
Blend, comparing age (Figure S1)	One curve for all datasets (0.061)	2.529 (3, 106)	0.804 (0.727 to 0.977) young; 0.887 (0.831 to 0.950) old	0.545 (0.727 to 0.977) young; 0.634 (0.556 to 0.719) old	0.089 (0.031 to 0.188) young; 0.114 (0.067 to 0.181) old	0.843 (0.796 to 0.899)	0.591 (0.530 to 0.657)	0.104 (0.064 to 0.158)

Table S1. Statistical analyses of behavior experiments. Related to Figure 1 and Figure S1.

The table includes comparisons of nonlinear regression models when fitting separate curves to each dataset or a single curve to all datasets. The preferred model was determined using the extra sum-of-squares F test. Logistic growth functions (Y=YM*Y0/((YM-Y0)*exp(-k*x)+Y0) were fit to all datasets except for the vehicle control, where a quadratic function was fit ($Y=B0+B1*X+B2*X^2$). See Data S1 for behavioral data.
